# Assessment of host–guest molecular encapsulation of eugenol using β-cyclodextrin

**DOI:** 10.3389/fchem.2022.1061624

**Published:** 2023-01-09

**Authors:** Camila Auad Beltrão de Freitas, Clauber Henrique Souza Costa, Kauê Santana da Costa, Simone Patrícia Aranha da Paz, José Rogério A. Silva, Cláudio Nahum Alves, Jerônimo Lameira

**Affiliations:** ^1^ Laboratório de Planejamento e Desenvolvimento de Fármacos, Instituto de Ciências Exatas e Naturais, Universidade Federal do Pará, Belém, Pará, Brazil; ^2^ Laboratório de Simulação Computacional, Instituto de Biodiversidade, Universidade Federal do Oeste do Pará, Unidade Tapajós, Santarém, Pará, Brazil; ^3^ Laboratório de Caracterização Mineral, Universidade Federal do Pará, Belém, Pará, Brazil

**Keywords:** repellents, nanoencapsulation, eugenol, molecular dynamics, molecular modeling

## Abstract

Eugenol is a natural compound with well-known repellent activity. However, its pharmaceutical and cosmetic applications are limited, since this compound is highly volatile and thermolabile. Nanoencapsulation provides protection, stability, conservation, and controlled release for several compounds. Here, eugenol was included in β-cyclodextrin, and the complex was characterized through X-ray diffraction analysis (XRD) and Fourier-transform infrared spectroscopy (FTIR). Additionally, we used molecular dynamics simulations to explore the eugenol–β-cyclodextrin complex stability with temperature increases. Our computational result demonstrates details of the molecular interactions and conformational changes of the eugenol–β-cyclodextrin complex and explains its stability between temperatures 27°C and 48°C, allowing its use in formulations that are subjected to varied temperatures.

## Introduction

Mosquitos are the main vectors of viral diseases that manifest predominantly in tropical and subtropical regions of the world, such as dengue, yellow fever, zika, and chikungunya (Paixão et al., 2018; Higuera and Ramírez, 2019; Barreto-Vieira et al., 2020). The chemical protection conferred by repellents against mosquitos has been an effective alternative to prevent their contact with the human skin (Ray, 2015; Mapossa et al., 2021). Natural products remain an interesting source of new bioactive compounds with different applications (Rayan et al., 2017; Galúcio et al., 2019; Do Nascimento et al., 2020; Newman and Cragg, 2020; Santana et al., 2021), and these structures have been widely investigated as a repellent against mosquitos (Tabanca et al., 2016; da Costa et al., 2019a). However, these compounds have been reported to have a short shelf life, in part, due to their volatile nature (Kayaci et al., 2013; Tan et al., 2019; Beltrán Sanahuja and Valdés García, 2021). Eugenol (4-allyl-2-methoxyphenol) is a volatile and lipophilic phenolic natural compound belonging to the class of phenylpropanoid, and it is mainly found in the essential oils of plants. Eugenol can be also produced synthetically by the allylation of guaiacol with allyl chloride (Moyer et al., 2002; Kuskoski et al., 2003). Eugenol is responsible for clove aroma, and it is well known for to its wide range of biological activities, such as antibacterial, antioxidant, anesthetic, and anti-inflammatory (Jaganathan et al., 2011; Kamatou et al., 2012; Roth-Walter et al., 2014; Xu et al., 2016; Mateen et al., 2019; Methods, 2021). The U S Food and Drug Administration also considers eugenol as a safe food additive for human use (Kamatou et al., 2012; El-Saber Batiha et al., 2020), and studies have demonstrated that eugenol has efficient repellent activity against different mosquitos species, such as *Aedes aegypti* (Miot et al., 2011; Afify and Potter, 2020), *Culex quinquefasciatus* (Afify et al., 2019), and *Anopheles gambiae* (Lupi et al., 2013; Thomas et al., 2017).

Several experimental studies have reported the formation of inclusion complexes of eugenol with encapsulating agents, such as β-cyclodextrin, to reduce the undesirable effects (localized irritation of the skin and allergic contact dermatitis), increase its aqueous solubility, and prolong its biological activity (Yang and Song, 2005; Abarca et al., 2016; Gong et al., 2016; Kfoury et al., 2018; de Freitas et al., 2021). β-cyclodextrin (cyclohepta-amylose) is a cyclic oligosaccharide formed by D-glucose monomers which are produced by the enzymatic degradation of starch (Szejtli, 1998; Wüpper et al., 2021), and it is particularly interesting for the encapsulation of volatile compounds, thus representing a viable and efficient strategy to retain and modulate the release of volatile and hydrophobic compounds (Abarca et al., 2016; Kfoury et al., 2018; Zheng et al., 2020). Cyclodextrin inclusion complexation is widely used in food, cosmetics, agrochemical, and pharmaceutical industries to increase the stability of several volatile organic compounds due to its hydrophobic cavities and hydrophilic exterior (Loftsson and Brewster, 1996; Szejtli, 1998; Muthu Vijayan Enoch and Swaminathan, 2004; Enoch and Swaminathan, 2005; Xu et al., 2021), which creates a physical barrier between the nucleus and the shell materials (Anaya-Castro et al., 2017).

The α-, β-, and γ-cyclodextrins are subclasses of cyclodextrins widely used for nanoencapsulation, and they could be differentiated by the presence of 6, 7, and 8 glucopyranose units, respectively, that determine the size of their internal cavity (Szejtli, 1998; Saha et al., 2016; Jansook et al., 2018). These cyclodextrins have a truncated cone-shaped molecular form, and their hydrophobic cavities have a remarkable ability to form non-covalent inclusion complexes with a variety of compounds (Lee et al., 2020; Pena et al., 2022). During the formation of an inclusion complex, water molecules are displaced to the outside of the lipophilic cavity, due to the presence of new lipophilic guest molecules that induce a new equilibrium (Anaya-Castro et al., 2017). This water displacement and the formation of a stable complex depends on the binding forces present in the inclusion complex (e.g., hydrophobic interactions, van der Waals attractions, hydrogen bonds, and electrostatic interactions) and temperature (Alvira, 2018; Lee et al., 2020).

Temperature is an important variable to assess the stability of the inclusion complexes, and understanding its influences on the formation of intermolecular interactions and mass loss is crucial to the experimental tests that evaluate the repellent efficiency (Kayaci and Uyar, 2011; Abarca et al., 2016; Celebioglu et al., 2018). Several studies have performed computational analyses to investigate the formation of inclusion complexes formed between the oligosaccharides and the natural products (de Sousa et al., 2016; Mustafa et al., 2021; Rezaeisadat et al., 2021). Similarly, experimental studies have investigated the formation of these complexes between eugenol and its derivates with β-cyclodextrin (Alvira, 2018; Joardar et al., 2020) and have identified a slower controlled release of eugenol at elevated temperatures, such as 50°C, 75°C, and 100°C (Kayaci et al., 2013; Celebioglu et al., 2018).

In the present study, we analyzed the eugenol–β-cyclodextrin inclusion complex through X-ray diffraction analysis (XRD) and Fourier-transform infrared spectroscopy (FTIR) and its chemical stability and binding affinity using molecular dynamics (MD) simulations and binding free energy calculations, respectively. The representative structures of the analyzed systems are shown in Supplementary Figure S1.

## Materials and methods

### Chemical reagents

The eugenol (CAS: 97-53-0, medium molecular weight: 164.2 g/ml, and purity: 99%) and the commercial β-cyclodextrin (CAS: 7585-39-9, medium molecular weight: 1,134.98 g/ml, and purity: 97%) were obtained from Sigma Aldrich Laboratory (São Paulo, Brazil).

### Synthesis of inclusion complexes

The inclusion complex was formed through co-precipitation and solvent evaporation (Ayala-Zavala et al., 2008), in which the hydroalcoholic solution of β-cyclodextrin was incorporated into an alcoholic solution of eugenol to obtain molar ratios 1:1, 2:1, and 2:3 in duplicate, to compare and analyze the influence of concentration molars in the final product of the complexes. The physical mixture was obtained by maceration, in grade and pistil, until the formation of a paste and homogenized for 15 min; then, it was allowed to rest for 24 h in an isolated environment, dried at 50°C for 12 h, and stored as other inclusion complexes.

### Characterization of the inclusion complexes

#### X-ray diffraction

The measurements were recorded in a divergent beam diffractometer (model: Empyrean from PANalytical) with a θ–θ goniometer, ceramic X-ray tube sealed with a cobalt anode, monochromatic radiation of Co-Kα1 (λ = 1.789 ″A"), long fine focus 1800W, and a Fe kβ filter. The detector PIXel3D 2 × 2 area was used with an active length of 3.3473° (2θ–2Theta) and 255 active channels. The following instrumental conditions were applied in the analyses: voltage of 40 kV and current of 40 mA, solar encapsulation slits of 0.04°rad (in the incident and diffracted beams), 2°–80° (2θ) sweep range, and 0.04° step size in 2θ with 1s time/step in continuous scan mode. Phase identification was performed using PANalytical’s HighScore Plus 4.8.0 software.

Powder X-ray diffractometry is a useful method to confirm the formation of complex powder or microcrystalline states; therefore, the XRD technique is only applied to materials (solid-state matter). In the present study, it was applied to analyze the materials: free β-cyclodextrin (β-CD), eugenol–β-cyclodextrin (EG-β-CD) complexes, and physical mixture (PM).

### Fourier transform infrared spectrometry

The spectroscopic investigations were performed to identify the functional groups of the eugenol–β-cyclodextrin complex in the middle infrared spectral region—Middle-IR (4,000–400 cm^−1^)—using a Thermo Scientific Fourier transform infrared spectrometer (model: Nicolet iS50 FTIR), a KBr (potassium bromide) beam splitter, an IR source, and a KBr DTGS detector. The measurements were obtained by transmission with KBr pellets (0.15 g) + sample (0.002 g), with an average of 100 scans and a resolution of 8 cm^−1^. Data were acquired using OMNIC software. As a pre-treatment, the samples were dried at 105°C for 24 h.

### Molecular modeling studies

#### Molecular docking

To investigate the most stable conformation of eugenol in complex with β-cyclodextrin, we performed molecular docking using AutoDock Vina (Trott and Olson, 2009). This computational method allowed us to describe the molecular interactions of the eugenol with the internal cavity (lipophilic) and external surface (hydrophilic) of the β-cyclodextrin nanoparticle. Herein, we used the crystallographic structure of β-cyclodextrin (PDB code: 3EDJ) as the starting point to perform the simulations (Buedenbender and Schulz, 2009). We used the following spatial coordinates for the docking grid: X = 69.62, Y = 67.16, and Z = 40.77, with dimensions of X = 40, Y = 40, and Z = 40 Å. The docking simulations were performed with 10 runs, and a total of 10 conformations per compound were set to perform the docking. The formation of the intermolecular interactions, such as H-bond, π-interactions, and hydrophobic interactions, were analyzed using BIOVIA Discovery Studio (BIOVIA, 2017).

#### Molecular dynamics simulations

To perform the MD simulations, we selected the lowest energy structure of the eugenol/β-cyclodextrin complex obtained from the docking simulations. First, the atomic charges of eugenol were calculated using the restrained electrostatic potential atomic partial charges (RESP) protocol (Wang et al., 2000; Wang et al., 2004) using the Hartree–Fock method with the 6-31G* basis set (Bayly et al., 1993) available in the Gaussian09 program (Frisch et al., 2009). The carbohydrate force field Glycam06 (Kirschner et al., 2008) was used to treat β-cyclodextrin, and the general AMBER force field (GAFF) was used to treat the complex formed with the eugenol (Wang et al., 2004). The complex was solvated in a cubic water box using the TIP3P model (Jorgensen et al., 1983; Jorgensen et al., 1996), and the distance between the box wall and atoms of the system was set to 12.0 Å.

The geometry and the inter- and intra-atomic distances of hydrogen molecules, water molecules, and the eugenol–β-cyclodextrin complex were optimized in seven minimization steps using 100,000 cycles of steepest descent and the conjugate gradient method (Hestenes and Stiefel, 1952). The β-cyclodextrin–eugenol complex was investigated in four different temperatures: 27°C, 38°C, 48°C, and 58°C. In the MD simulations, the systems were heated to their final temperature (300 K) to equilibrate the density and maintain the constant pressure (1 atm). The SHAKE algorithm (AndersenRattle, 1983) was applied for all hydrogen molecules of the analyzed systems. A total time of 50 ns of MD simulation was performed using NPT ensemble.

## Results and discussion

### Experimental characterization of inclusion complexes

#### X-ray diffraction (XRD)

**FIGURE 1:**
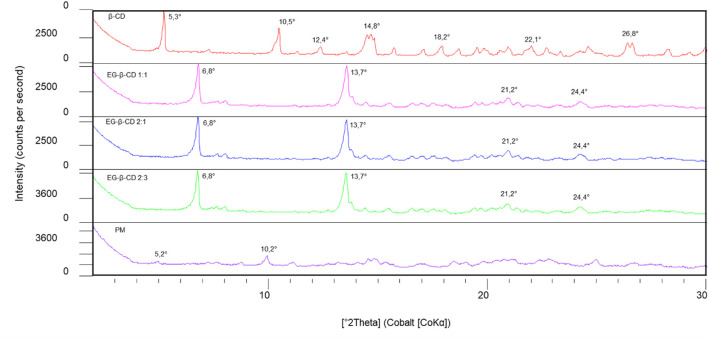
Diffraction patterns of free β-cyclodextrin (β-CD), inclusion complex of eugenol–β-cyclodextrin in a 1:1 ratio, inclusion complex of eugenol–β-cyclodextrin in a 2:1 ratio, inclusion complex of eugenol–β-cyclodextrin in a 2:3 ratio, and physical mixture (PM) were analyzed using the diffractograms.

The diffraction pattern of the β-cyclodextrin–eugenol complexes in different stoichiometric proportions showed a similar trend, demonstrating only some different intensities between the 2:3 ratio and the others, in which the peaks are presented in greater intensity, in 3600 counts (Figure 1). However, all analyzed complexes show considerable differences when compared to the diffraction pattern of free β-cyclodextrin and the physical mixture (Figure 1). The free β-cyclodextrin diffractogram presents many Bragg reflections, highlighting the high-intensity peaks in °2θ (CoKα): 5.3°, 10.5°, 12.4°, 14.8°, 18.2°, 22.1°, and 26.8°, as observed in studies carried out with β-cyclodextrin and other guests (Wang et al., 2011; Gong et al., 2016; Yang et al., 2016; Jiang et al., 2019), having as characteristic peak the angle °2θ at 5.3° provided by the database of software used.

We found a significant difference between the diffractograms of free β-cyclodextrin and the β-cyclodextrin and eugenol complexes, indicating the occurrence of encapsulation and the interaction between eugenol and β-cyclodextrin, given a reordering in the crystal structure, by the disappearance of peaks in 10.5° and 12.4 °2θ (CoKα) and contraction of the unit cell with decreasing dhkl, that is, by increasing the angle, as observed for 6.8, 13.7, 21.2° and 24.4 °2θ (CoKα), data that corroborate the results previously found (Yang and Song, 2005; Abarca et al., 2016; Dos Passos Menezes et al., 2017), which may be associated with changes in the molecular organization of β-cyclodextrin during the production of complexes.

**FIGURE 2:**
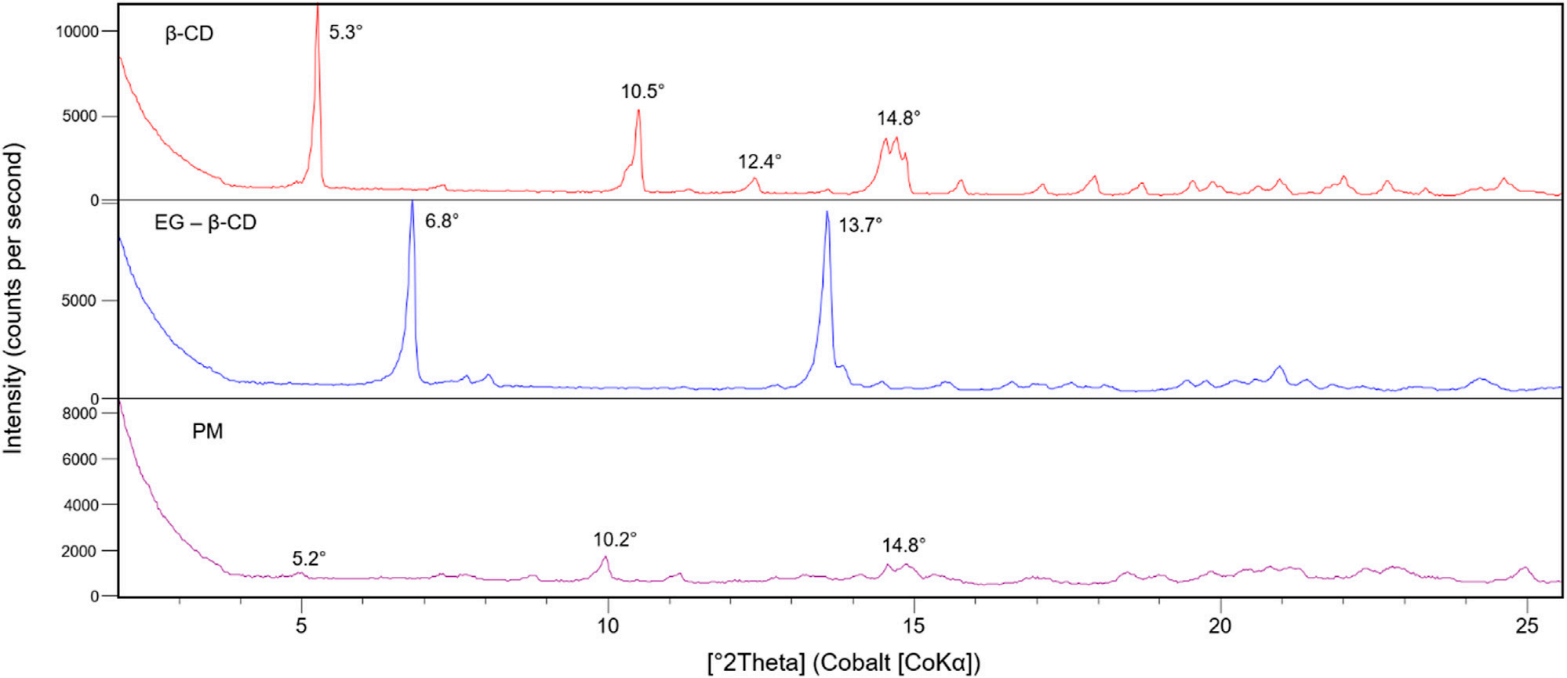
Diffraction patterns of free β-cyclodextrin (β-CD), eugenol–β-cyclodextrin (EG-β-CD) complex, and physical mixture (PM) were analyzed using the diffractograms.

The peak shifts indicate that the diffraction pattern of free β-cyclodextrin was altered when the eugenol was incorporated into the host molecule cavity. In studies that present the physical mixture diffractogram between eugenol and β-cyclodextrin, crystalline peaks of β-cyclodextrin were detected at a lower intensity, indicating that there was no marked difference in the crystalline form of β-cyclodextrin (Abarca et al., 2016; Gong et al., 2016), as seen in Figure 2. Furthermore, it is also important to note that the peak intensities in the eugenol–β-cyclodextrin complex were attenuated in relation to the same peaks in the free β-cyclodextrin spectrum, indicating greater structural disorder or loss in the degree of crystallinity for the complex (Figure 2). This fact is attributed to the rapid precipitation of the complex during preparation, which makes regular crystal growth insufficient (Yang and Song, 2005).

In this analysis, the diffractograms of the pure species were compared with the values obtained of the complex (Cao et al., 2005). The differences obtained from the analyses, such as the appearance or disappearance of peaks or changes in relative intensities, evidenced the formation of the inclusion complex since the principle of the complexation is associated with an increase in the degree of amorphization of the substances involved in the formation of the complex in the solid-state (Ribeiro et al., 2008; Gao et al., 2020).

#### Fourier transform infrared spectrometry (FTIR)

**FIGURE 3:**
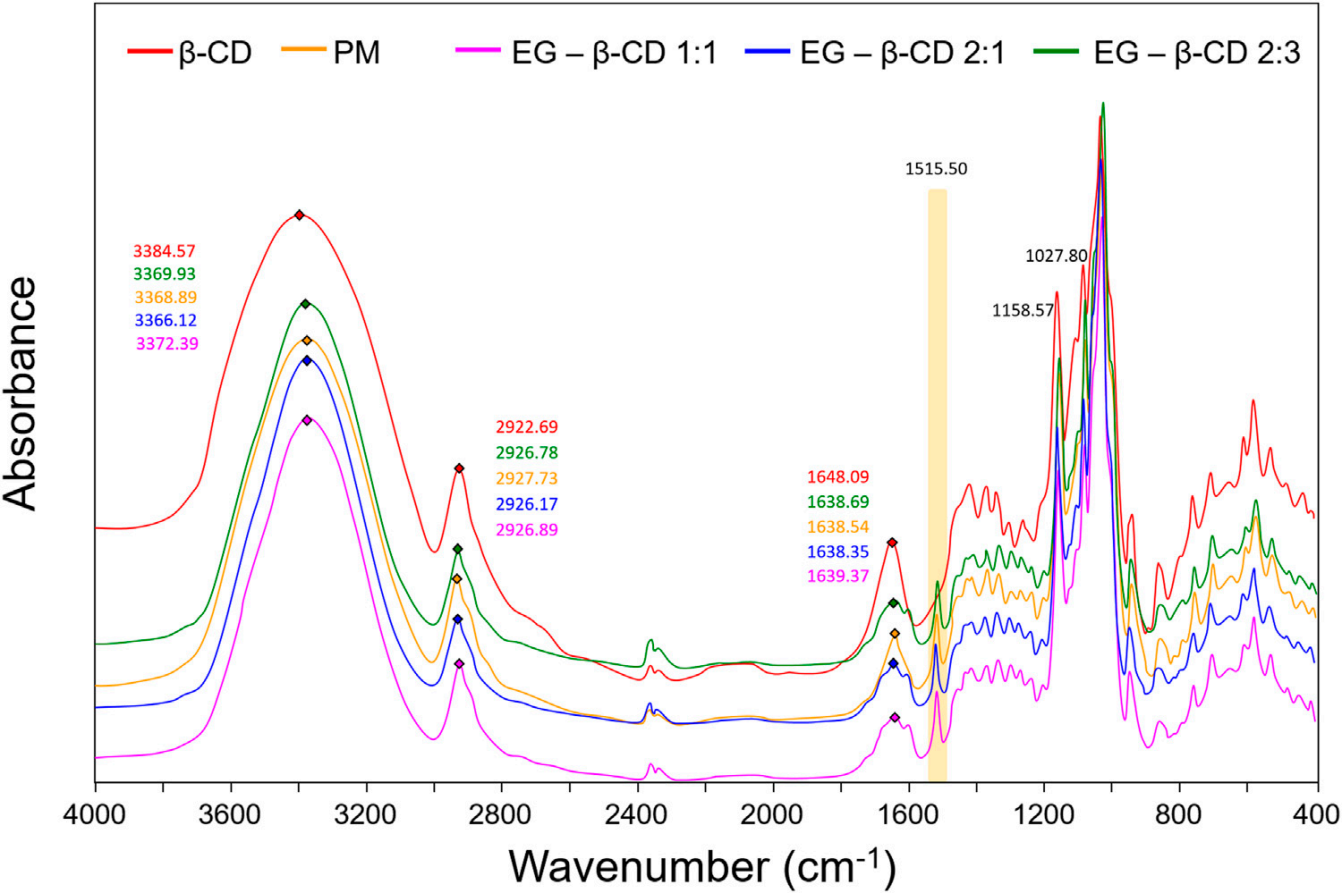
Comparison between the FTIR spectra for the β-cyclodextrin, physical mixture of eugenol and β-cyclodextrin, inclusion complex of eugenol–β-cyclodextrin in a 1:1 ratio, inclusion complex of eugenol–β-cyclodextrin in a 2:1 ratio, and inclusion complex of eugenol–β-cyclodextrin in a 2:3 ratio.

The FTIR technique is a very helpful tool to prove the interaction of both guest and host molecules in their inclusion complexes in a solid phase (Singh et al., 2010). Figure 3 shows the FTIR spectra for 1) β-cyclodextrin, 2) physical mixture of eugenol and β-cyclodextrin, 3) inclusion complex of eugenol–β-cyclodextrin in a 1:1 ratio, 4) inclusion complex of eugenol–β-cyclodextrin in a 2:1 ratio, and 5) inclusion complex of eugenol–β-cyclodextrin in a 2:3 ratio. The spectra bands which characterize the absorption regions of β-cyclodextrin are associated with the stretches, referring to the symmetric and asymmetric deformation of the hydroxyl group (OH) in the range of 3,600–3,000 cm^−1^, that showed the characteristic bands of primary and secondary OH groups in 3384.57 cm^−1^.

**FIGURE 4:**
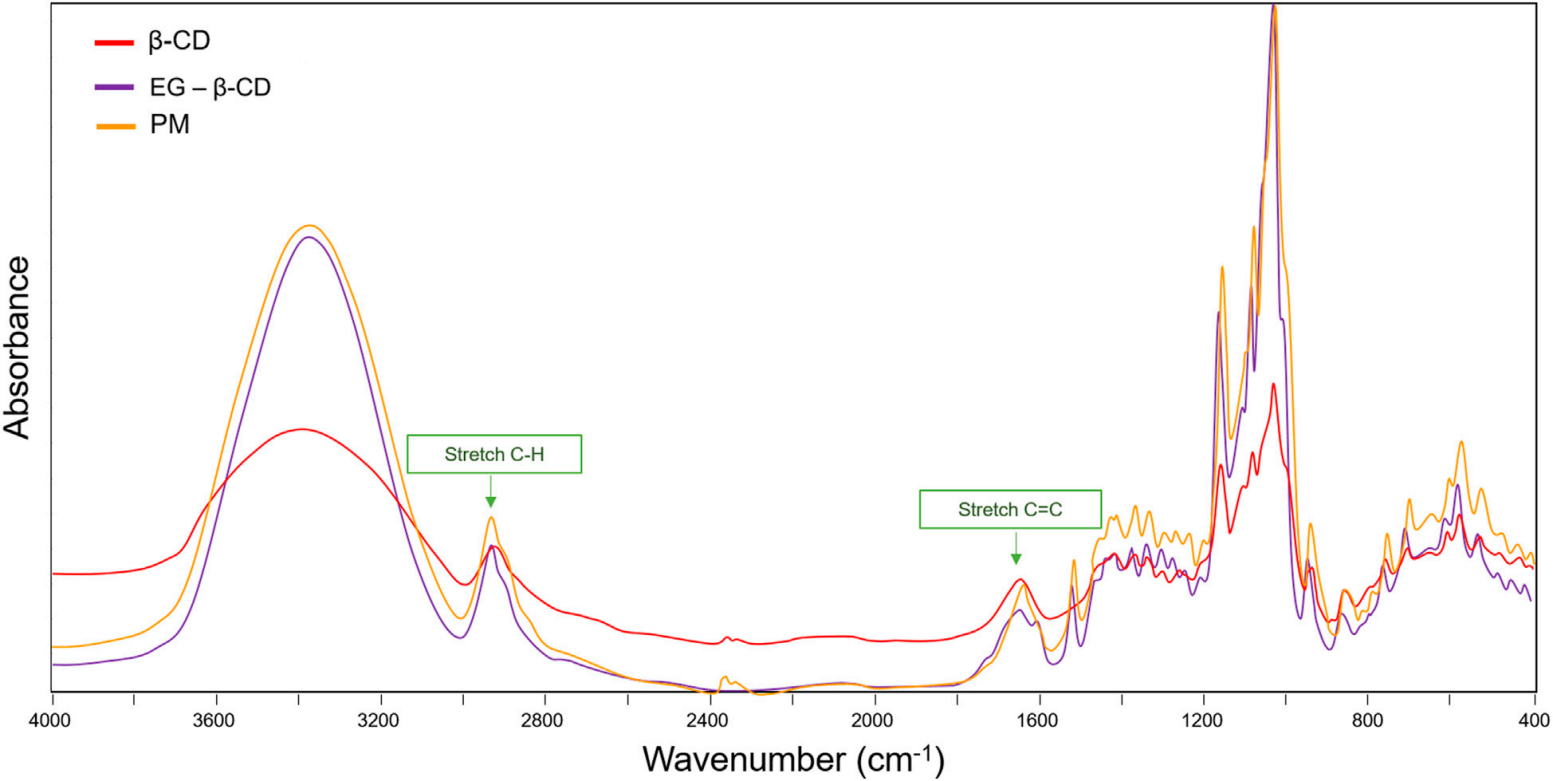
Comparative spectra between free β-cyclodextrin, inclusion complex, and physical mixture.

We also observed the CH bands of the β-cyclodextrin ring and methyl groups in the range of 2,940–2,840 cm^−1^. These results were also described in previous studies (Celebioglu et al., 2018; Hadian et al., 2018), and the bands between 1,700 cm^−1^ and 1,600 cm^−1^ are associated with HOH bonds which are also abundant in the compound (Abarca et al., 2016). IR spectra are particularly sensitive to the presence of water; therefore, the spectral region of interest presents this contribution assigned to the HOH bending mode (Venuti et al., 2015). In addition, the spectra display the OH bending vibration in the range of 1,030–1,015 cm^−1^ and C-O-C stretch, between 1,159 and 1,143 cm^−1^. These elongation vibrations were described previously in the literature (Celebioglu et al., 2018; Gao et al., 2020). A broad hydroxyl band of pure β-cyclodextrin spectrum (Figures 3, 4) shows the maximum absorption at 3,384.57 cm^−1^, found in the FTIR spectrum of the inclusion complexes which is a good indication of their formation due to the stretching vibrations of the different β-cyclodextrin OH groups (Yang and Song, 2005; Kayaci et al., 2013; Celebioglu et al., 2018; Briñez-Ortega et al., 2020).

Table 1 shows some increase and decrease in intensity changes due to the insertion of the part ring into the electron-rich cavity of β-cyclodextrin. Some bands showed little or no changes in the band upon complexation, implying that the inclusion of guest molecules in the CD cavity does not affect this vibrational mode. Although the spectrum of the inclusion complex appears almost similar to that of β-cyclodextrin alone, these results indicate the formation of the inclusion complex due to the weak interactions when partial inclusion of the ligand occurs (Stepniak et al., 2015; Vestland et al., 2015).

**TABLE 1:** Comparison between the intensity of free β-cyclodextrin, inclusion complexes, and physical mixture.

Functional group	Wavenumber (cm^−1^)				
			
	β-CD	Inclusion complex 1:1	Inclusion complex 2:1	Inclusion complex 2:3	PM
ν[OH] symmetric and antisymmetric	3,384.57	3,372.39	3,366.12	3,369.93	3,368.89
ν[CH]	2,922.69	2,926.89	2,926.17	2,926.78	2,927.73
ν[C–O–C]	1,158.57	1,156.85	1,156.64	1,156.92	1,157.10
ν[O–H] bending vibration	1,027.80	1,027.42	1,028.06	1,027.57	1,028.02

In relation to the presence of eugenol in the composition of the complex, it can be proven by the presence of absorption regions at 1515.50 cm^−1^ (Figure 3), related to the C=C bonds of the aromatic ring of the compound that usually appears in the region between 1650 and 1250 cm^−1^ corresponding to the vibration of the C=C groups and CH flexion of the alkene/aromatic groups of the eugenol (Yang and Song, 2005; Kayaci et al., 2013; Celebioglu et al., 2018). Additionally, frequencies between 1,200 cm^−1^ and 1,000 cm^−1^ are related to the presence of CO stretches (Yang and Song, 2005; Scremin et al., 2018). In addition, for eugenol, the characteristic bands are found in the ranges of 3,380–3,360 cm^−1^ due to the axial stretching of the OH group (Rodríguez et al., 2021). Therefore, the results presented previously indicate the efficiency of the encapsulation process of the eugenol molecules by the β-cyclodextrin (Wang et al., 2011).

Generally, the comparative analysis of the physical mixture with the inclusion complex (Figure 4) presents the simple sum of the β-cyclodextrin and ligand bands (Zheng et al., 2020). A similar result was observed in the spectra. In the region of the absorption bands of the C–H stretches, we noted an overlap of the complex bands with that of β-cyclodextrin, without changes in the wavenumbers of the pure components. Similar results were noted in the region between 1,700 cm^−1^ and 1650 cm^−1^, where stretch bands C=C with subtle deformation were observed. These results suggest that the simple mixture of the two components in the solid phase is not enough to prove the formation of the inclusion complex, once the shape, intensity, and position of the peaks vary. These observations, combined with the results obtained by XRD, can be considered strong evidence of the formation of the inclusion complex.

### Molecular modeling analyses

#### Molecular interactions and formation of host–guest complex model

Molecular modeling analyses have been widely applied to assess the conformational, magnetic, and electronic properties of molecules (de Castro et al., 2014; de Sousa et al., 2016; de Souza Farias et al., 2021; Mustafa et al., 2021). Here, these computational analyses of the host–guest interactions were performed to better understand the formation of the eugenol–β-cyclodextrin complex and to provide additional insights into the complex model, especially when it is subjected to different temperatures in an aqueous solution.

**FIGURE 5:**
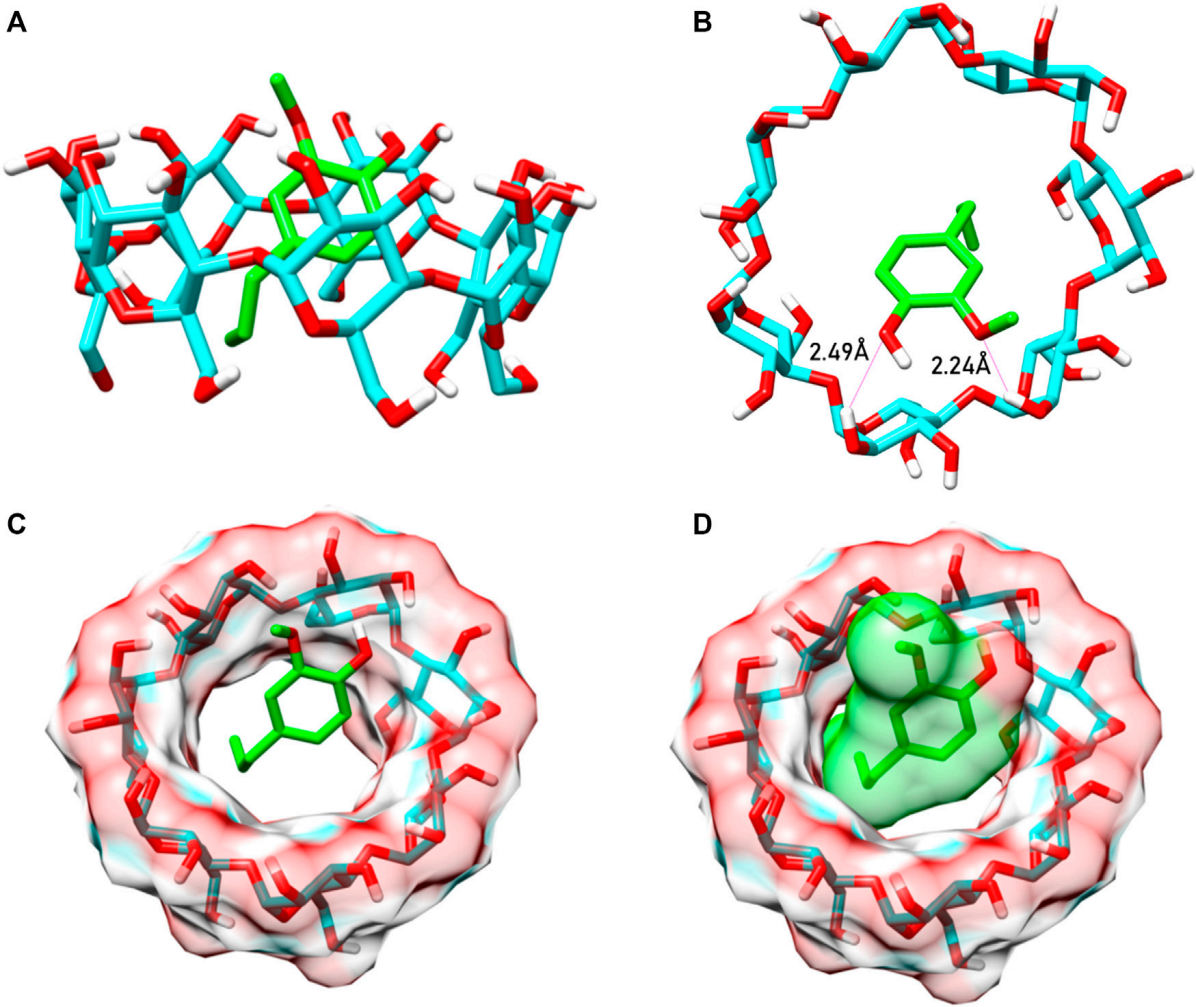
Inclusion complex formed between the eugenol (green) and the β-cyclodextrin (cyan). **(A)** 3D structure of BCD with eugenol forming the complex. **(B)** Exerting hydrogen bonds with the main electronegative group structure of EG. **(C)** EG (in green) inside the BCD cavity. **(D)** Eugenol with VDW surface, demonstrating binding in the BCD cavity.

The molecular analyses of the inclusion complex using molecular docking demonstrated that eugenol predominately had van der Waals interactions with the interior cavity of the β-cyclodextrin. Previous studies demonstrated that the geometric optimization of eugenol using density functional theory led eugenol to project its structure onto the external surface of the host (Mahboub, 2014; Chowdhry et al., 2015). Figure 5 shows the final conformation acquired by eugenol when complexed with β-cyclodextrin, obtained from molecular docking. The complex showed binding energy equal to −4.0 kcal mol^−1^, and two hydrogen bond interactions were formed in the complex which contributed to the stability of eugenol in the inclusion complex. These molecular findings agree with the results reported previously (Joardar et al., 2020).

#### Molecular dynamics simulations of eugenol–β-cyclodextrin complex at different temperatures

**FIGURE 6:**
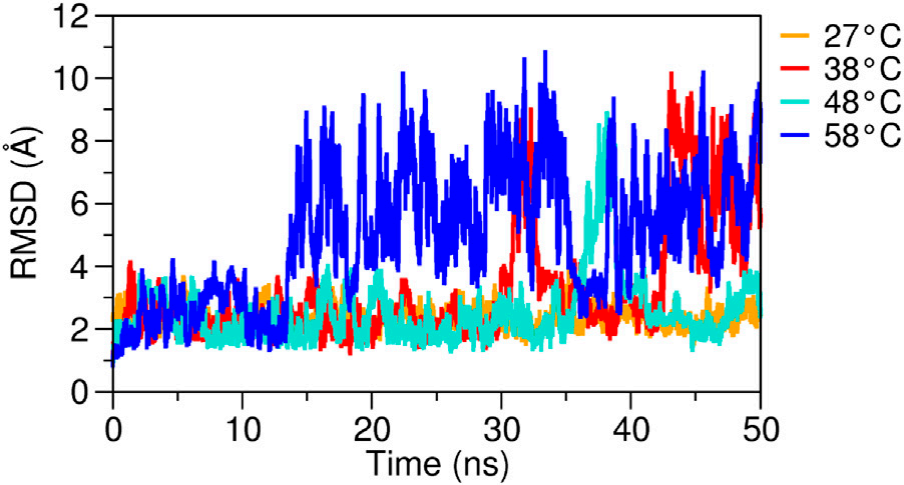
RMSD plots of the eugenol–β-cyclodextrin complex were evaluated at different temperatures. Complex analyzed over 50 ns of MD simulation at 27°C, 38°C, 48°C, and 58°C.

Experimental thermal analyses, in general, reveal marked structural differences between isolated molecules and the inclusion complexes. In addition, these analyses exhibit a typical sharp melting endotherm at temperatures over 250° C, which is indicative of the anhydrous and crystalline state of the analyzed molecules; it also exhibits effects regarding their dehydration and degradation process (Seo et al., 2010; Piletti et al., 2017; Rodríguez et al., 2021). Since previous studies have identified a slower controlled release of eugenol at elevated temperatures, such as 50°C, 75°C, and 100°C (Kayaci et al., 2013; Piletti et al., 2017; Celebioglu et al., 2018), we analyzed the eugenol–β-cyclodextrin complex using a gradual temperature increase to evaluate the behavior and stability of the complex in aqueous solution, which is also compatible with its applications in repellent formulations (Mapossa et al., 2021). Computationally, we demonstrated that when the temperature is increased, it directly affects the interaction and stability of the complex (see RMSD plot, Figure 6A).

Between temperatures 27°C and 48°C, the complex showed stability, and eugenol maintained its interactions. However, we noted that for the temperature of 58°C, the RMSD plot showed high fluctuations (Figure 6), and eugenol was shown to be unstable in the complex, thus losing some of its interactions with the host and leaving the β-cyclodextrin cavity. Low stability of the inclusion complex can be attributed to poor interaction and orientation of eugenol inside the β-cyclodextrin cavity. These results demonstrate that the high temperatures impair the formation of the eugenol–β-cyclodextrin complex, reducing its interactions and leading to the departure of the eugenol from the complex. We conjecture that the complex will remain stable in solution when subjected to moderate temperatures due to the protection and stability provided by the β-cyclodextrin.

**FIGURE 7:**
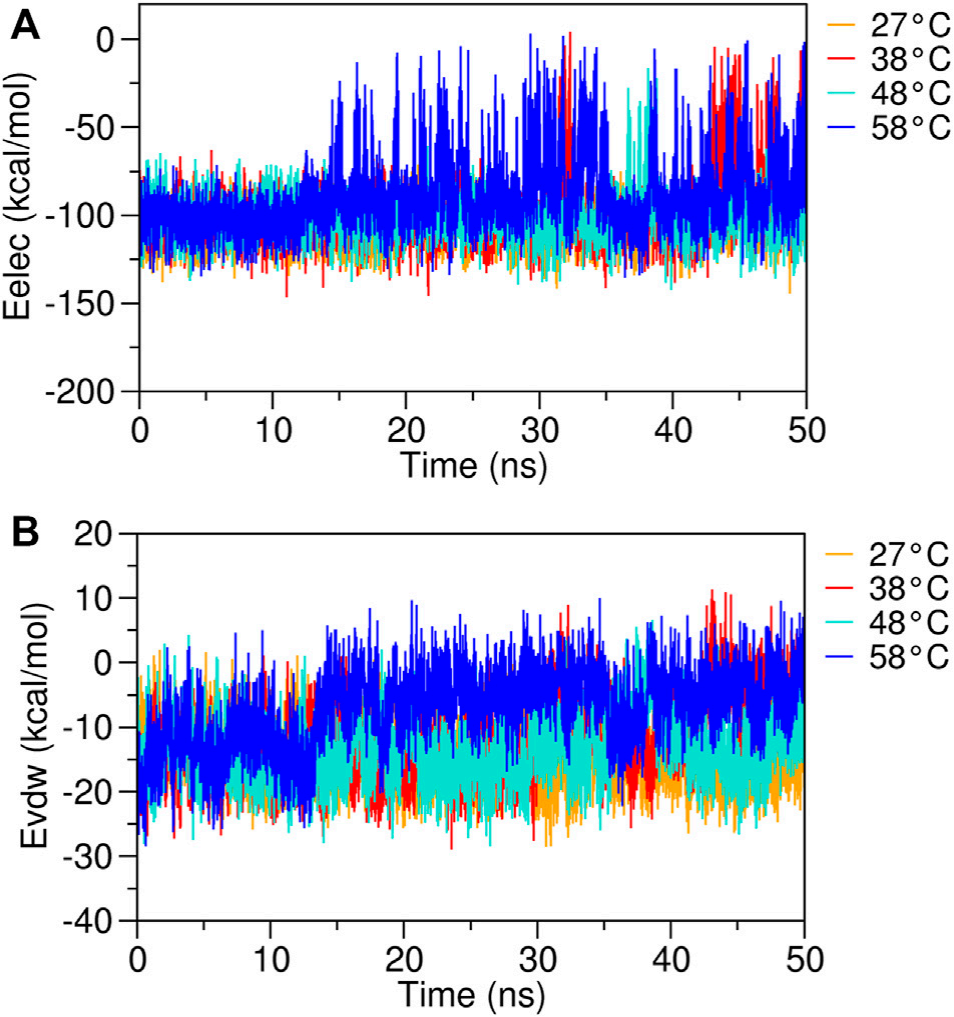
RMSD plots of the eugenol–β-cyclodextrin complex were obtained from different temperatures. **(A)** electrostatic energy (Eelec) and **(B)** van der Waals energy (Evdw) complex analyzed over 50 ns of MD simulation at 27°C, 38°C, 48°C, and 58°C.

Since the binding affinity depends on the molecular interactions formed between eugenol and the β-cyclodextrin surface and directly affects the temperature increase (Supplementary Figures S2, S3, S4), we investigated the β-cyclodextrin–eugenol complex using different temperatures. The electrostatic (E_elec_) and van der Waals (E_vdw_) energies of the analyzed systems (Figure 7) were calculated using the LIE implemented in the Cpptraj program (https://amberhub.chpc.utah.edu/lie/) (Åqvist et al., 2002; Brandsdal et al., 2003; Roe and Cheatham, 2013). At 20 ns and 30 ns of MD trajectory, the complex showed an E_elec_ value of approximately −100 kcal mol^−1^; however, with temperature increase, the energies tend to move toward values near zero. In contrast, the E_vdw_ increases with increasing temperature , thus indicating the presence of repulsion forces caused by the instability of the complex. Recently, a study demonstrated that the binding affinity in host–guest systems including β-cyclodextrin may be estimated with a root mean square error <1.5 kcal mol^−1^ from the experimental results using the LIE method (Montalvo-Acosta et al., 2018), which is closely related to the error of <1 kcal mol^−1^ found in the experimental values obtained in the prediction of the relative binding affinity for a vast range of protein–ligand systems (Gapsys et al., 2020).

**FIGURE 8:**
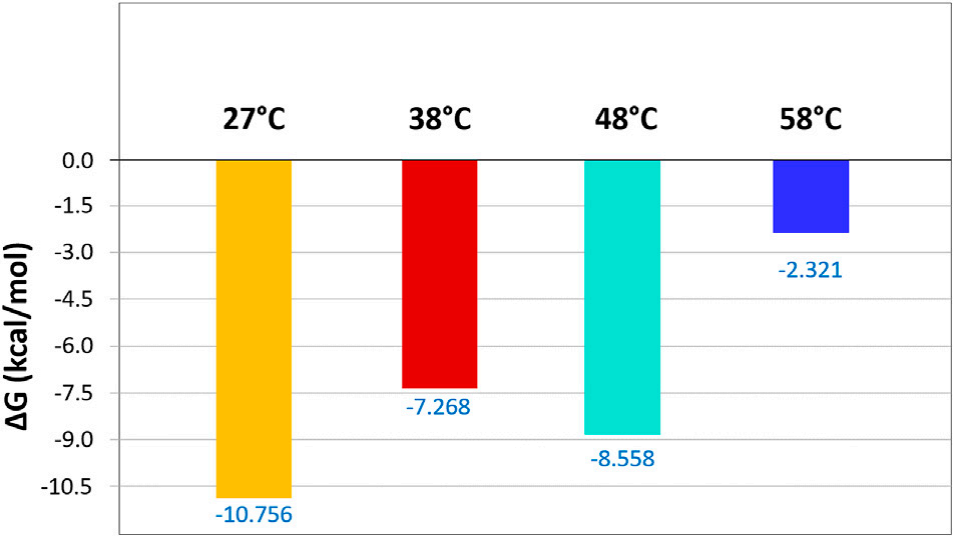
Binding free energies (kcal mol^−1^) were calculated using the MM/GBSA method for the eugenol–β-cyclodextrin complex. The inclusion complex was analyzed at different temperatures (27°C, 38°C, 48°C, and 58°C).

Additionally, the binding free energy was calculated using the MM/GBSA method for different temperatures, considering the total complexation time (Figure 8). The LIE and MM/GBSA are both end-point methods widely applied to investigate the binding free energy of biomolecular complexes (Cardoso et al., 2021; Costa et al., 2021; da Costa et al., 2019b; de Oliveira et al., 2020; Fonseca et al., 2020), and their results tend to show a trend similar to the energy values obtained from the experimental methods (Hansson et al., 1998; Aqvist and Marelius, 2001; Zhu et al., 2017; Rifai et al., 2020). The energy at room temperature (27°C) showed a highly stable complex, with eugenol demonstrating a high binding affinity with the β-cyclodextrin. However, with the increase of the temperature, the complex stability is affected and we have a decrease in the free energy of the analyzed systems, thus reducing the affinity of eugenol and causing its departure from the complex. This result indicates that the eugenol–β-cyclodextrin complex is stable at moderate temperatures and guarantees the permanence of the molecule inside the β-cyclodextrin with stronger binding.

## Conclusion

Here, we obtained eugenol–β-cyclodextrin inclusion complexes through co-precipitation and solvent evaporation. Then, the inclusion complex was characterized using the X-Ray diffraction and Fourier transform infrared spectroscopy, confirming the formation of the host–guest inclusion complex. Additionally, our computational analyses demonstrated that the eugenol–β-cyclodextrin complex remains stable between temperatures 27°C and 48°C. In contrast, high temperatures impair the formation of the eugenol–β-cyclodextrin complex, reducing its interactions and leading to its premature departure from the complex which is consistent with controlled release of the repellent.

## Data Availability

The original contributions presented in the study are included in the article/Supplementary Material; further inquiries can be directed to the corresponding author.
