# Supplementary material for: Assessment of host–guest molecular encapsulation of eugenol using β-cyclodextrin
Source: Front Chem. 2023 Jan 9;10:1061624. doi: 10.3389/fchem.2022.1061624 (PMC9868465; doi:10.3389/fchem.2022.1061624)
Supplement: Supplementary file 1 [file DataSheet1.docx]

SUPPORTING INFORMATION

**Assessment of Host-guest Molecular Encapsulation of Eugenol using β-cyclodextrin**

Camila Auad Beltrão de Freitas^1^, Clauber Henrique Souza Costa^1^, Kauê Santana da Costa^2^, Simone Patrícia Aranha da Paz^3^, José Rogério A. Silva^1^, Cláudio Nahum Alves^1^ and Jerônimo Lameira^1*^

^1^Laboratório de Planejamento e Desenvolvimento de Fármacos, Instituto de Ciências Exatas e

Naturais, Universidade Federal do Pará, Belém, Pará, Brazil, ^2^Laboratório de Simulação Computacional,

Instituto de Biodiversidade, Universidade Federal do Oeste do Pará, Unidade Tapajós, Santarém, Pará,

Brazil, ^3^Laboratório de Caracterização Mineral, Universidade Federal do Pará, Belém, Pará, Brazil

**KEYWORDS:** repellents, nanoencapsulation, eugenol, molecular dynamics, molecular modeling


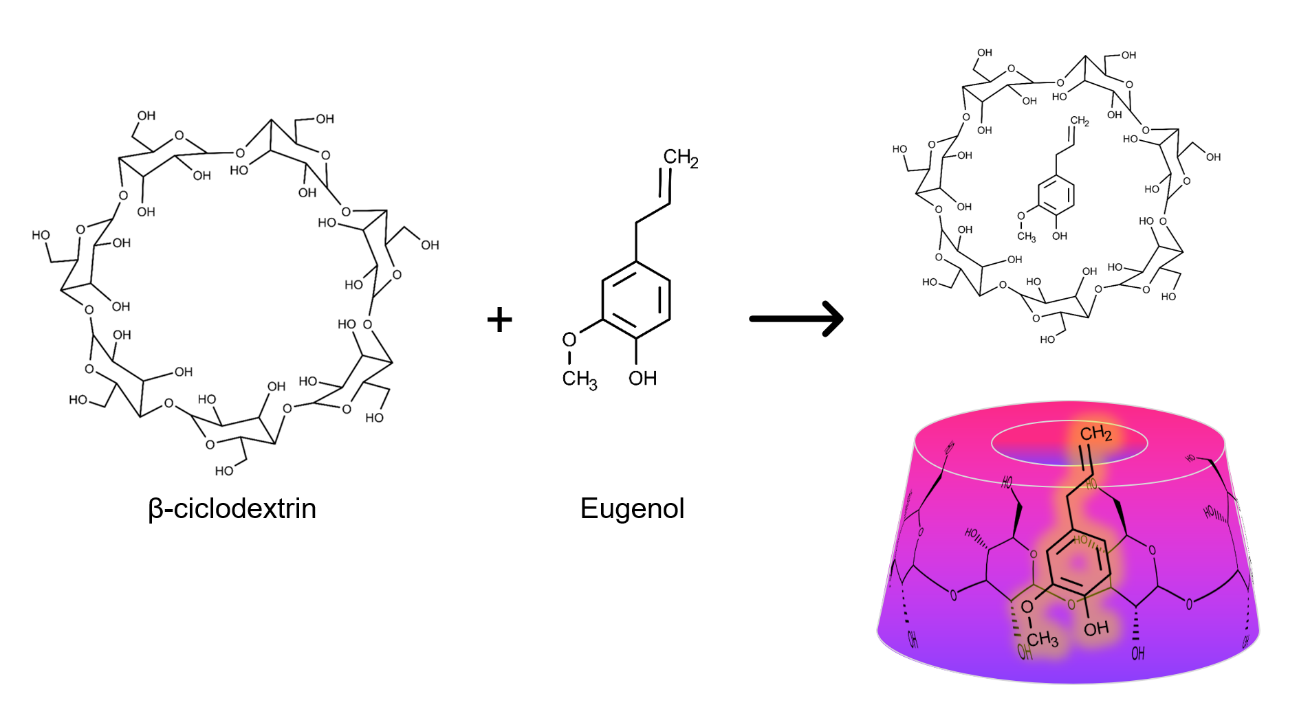


Figure S1. The representative structures of the analyzed systems, the formation eugenol-β-cyclodextrin complex.

**
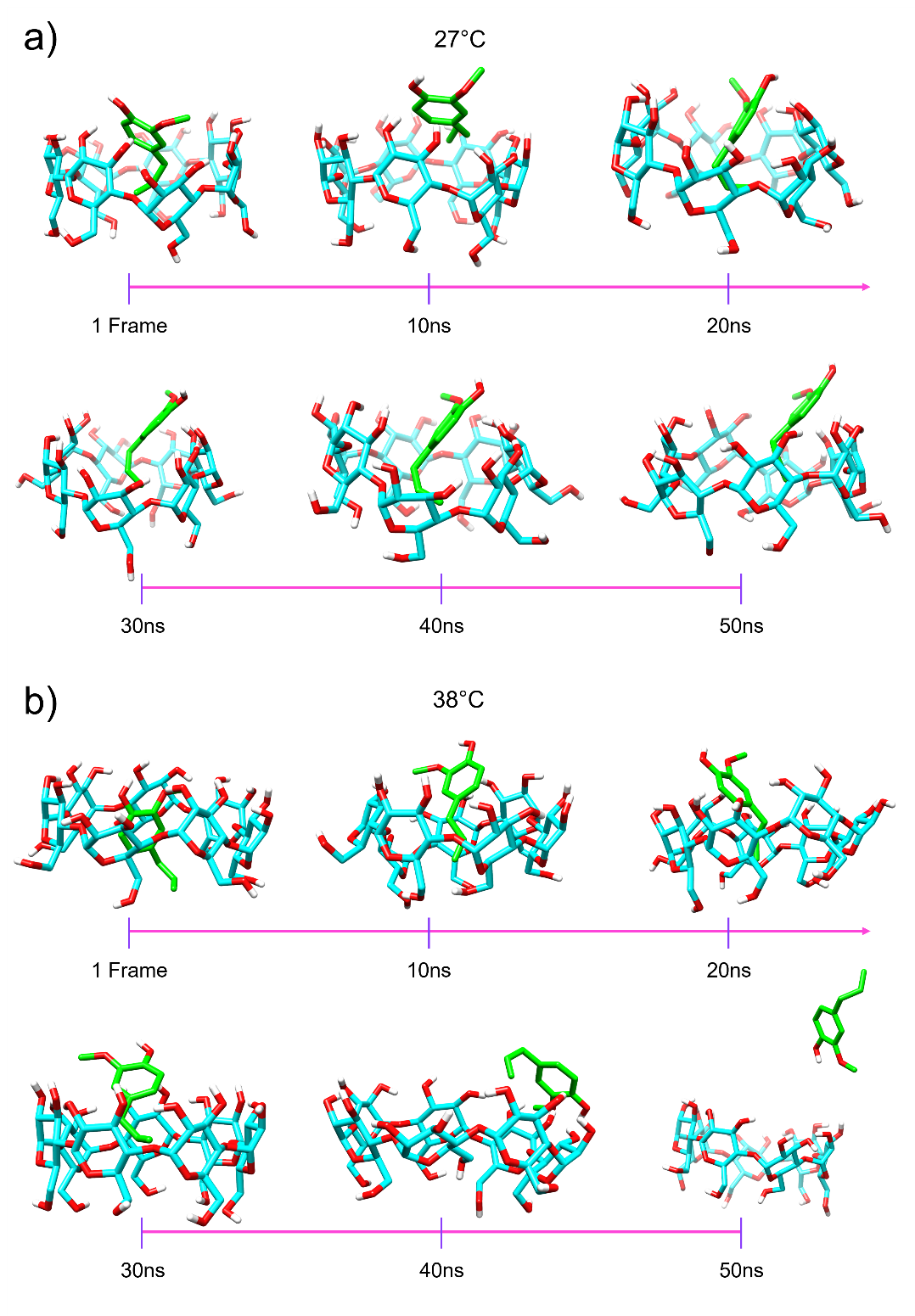
**

Figure S2. A) Conformations of the eugenol-β-cyclodextrin complex were obtained over the MD at a temperature of 27 ºC. B) Conformations of the inclusion complex obtained over the MD at a temperature of 38 ºC.


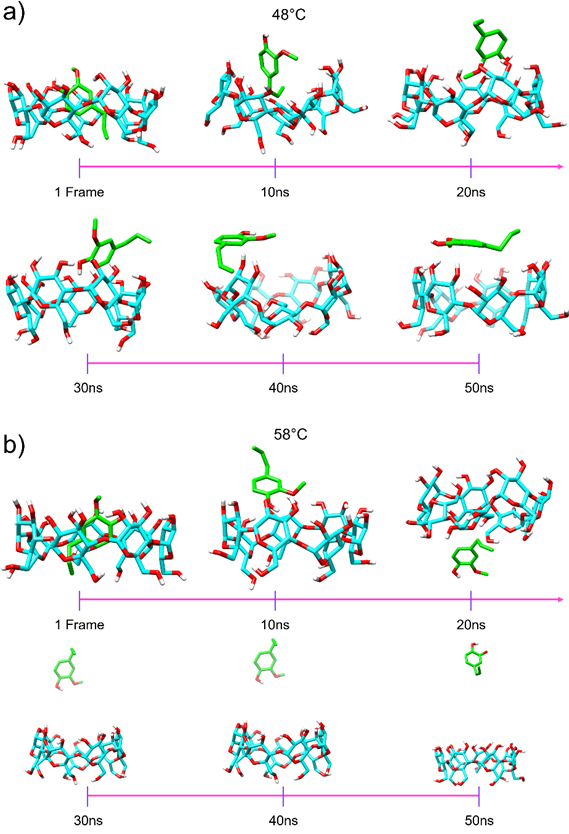


Figure S3. a) Conformations of the eugenol-β-cyclodextrin complex obtained over the MD at a temperature of 48°C. b) conformations adopted by eugenol and β-cyclodextrin during 50ns of DM at a temperature of 58°C.
